# The effect of parenting styles on Chinese undergraduate nursing students’ academic procrastination: the mediating role of causal attribution and self-efficacy

**DOI:** 10.3389/fpsyg.2023.1167660

**Published:** 2023-07-11

**Authors:** Yuanyuan Li, Wanglin Dong, Haishan Tang, Xiajun Guo, Sijia Wu, Guangli Lu, Chaoran Chen

**Affiliations:** ^1^Institute of Nursing and Health, School of Nursing and Health, Henan University, Kaifeng, China; ^2^Institute of Business Administration, School of Business, Henan University, Kaifeng, China

**Keywords:** parenting style, academic procrastination, attribution style, self-efficacy, nursing students

## Abstract

**Background:**

Academic procrastination is common among college students, but there is a lack of research on the influencing mechanism of academic procrastination among nursing students. The purpose of this study was to explore the influence of parental rearing patterns on academic procrastination of nursing students, and the mediating role of causal attribution and self-efficacy.

**Methods:**

Using Parental Bonding Instrument, Aitken Procrastination Inventory, Multidimensional Multi-Attribution Causality Scale and General Self-Efficiency Scale, the data of 683 nursing undergraduates from two universities in China were collected. Moreover, path analysis for structural equation modeling via AMOS 26.0 to evaluate mediation path model.

**Results:**

Positive parenting style was negatively associated with academic procrastination (*r* = –0.350) and negative parenting style was positively associated with academic procrastination (*r* = 0.402). Positive parenting style directly or indirectly predicted academic procrastination through the mediating effect of internal attributional style (β = –0.10, 95% CI: –0.18 to –0.04) and self-efficacy (β = –0.07, 95% CI: –0.11 to –0.03), and this mediating effect accounted for 41.46% of the total effect. Positive parenting style directly or indirectly predicted academic delay through the mediating effect of external attributional style (β = 0.12, 95% CI: 0.07 to 0.17) and self-efficacy (β = 0.05, 95% CI: 0.03 to 0.08), and this mediating effect accounted for 42.5% of the total effect. In addition, causal attribution and self-efficacy of nursing students play a chain intermediary role between parenting style and academic procrastination.

**Conclusion:**

Parents should give students more care and autonomy and reduce control. In addition, educators should give students attribution training, which is helpful to improve students’ self-efficacy and reduce academic procrastination.

## 1. Introduction

Procrastination refers to unnecessary and harmful delays, meaning “leaving things for tomorrow”. Procrastination is always manifested in various small things, but accumulated over time can not only lead to poor academic performance but also lead to negative emotions that can affect personal development. Among college students, procrastination in academics is a common problem. For example, the academic procrastination rate of medical undergraduates on various academic tasks is between 13.8 and 49.9% (Madhan et al., 2012; Mortazavi et al., 2015). Nursing students are faced with heavy academic tasks, standardized examinations, clinical practice and complicated interpersonal relationships (Boardman, 2016; Wang et al., 2019; Melo et al., 2020), so nursing students are prone to academic procrastination. Continuous academic procrastination will not only affects the academic performance of nursing students, but also prevent them from obtaining the knowledge and skills to provide quality care for patients (Guo et al., 2019). Moreover, with a global shortage of nursing staff and the growing need for healthcare, it is urgent to foster more and higher-quality nursing students. Therefore, educators should make clear the causes of academic procrastination and formulate effective intervention measures to reduce academic procrastination, which will be conducive to cultivating more high-quality nursing talents.

### 1.1. Parenting styles, academic procrastination, and causal attribution

Parenting style is defined as a constellation of parents’ attitudes and behaviors toward children and an emotional climate in which the parents’ behaviors are expressed (Darling and Steinberg, 1993). Baumrind identified three styles of parental authority, including permissive, authoritarian, and authoritative, and classified them into positive and negative parenting styles with reference to the dimensions of parental responses and demands (Soysa and Weiss, 2014). In short, parenting styles typically fall somewhere between lax and overly punishing, with extremes in either direction defined as negative (Xu et al., 2017). Positive parenting styles are characterized by high levels of parental care (e.g., supportive and encouraging autonomy). In contrast, negative parenting styles are characterized by parental rejection and overprotection (e.g., heavy supervision and monitoring, coercion, and authoritarianism) (Lian et al., 2016; Chen et al., 2022). In recent years, empirical evidence consistently shows the importance of parents’ socialization to children’s adaptation, pointing out the influence of parents’ socialization on children’s psychosocial adaptation and academic achievement (Pinquart, 2016; Waterman and Lefkowitz, 2017), with this influence continuing until adulthood (Garcia et al., 2018). For example, in young adult children, differences in their adjustment and competence seem to be related to parenting during socialization years (Palacios et al., 2022). Positive parenting styles provide a safer and more stable atmosphere, which is conducive to the development of children’s healthy personality and the promotion of children’s education and social and economic progress. On the contrary, negative parenting can weaken a child’s personality, self-confidence and character, hinder personality development, foster maladaptive behaviors and possibly lead to academic procrastination (Tang et al., 2014; Khalid et al., 2019). Hence, students’ academic procrastination may be related to parenting style. Specifically, Ferrari and Olivette found a positive relationship between fathers who used an authoritarian parenting style and daughters who showed general procrastination, whereas daughters of fathers with authoritative parenting styles did not report this general procrastination tendency (Ferrari and Olivette, 1994). Previous studies have also found that high levels of procrastination are related to high levels of parental monitoring behavior (Hong et al., 2015) and punishment (Ma et al., 2011). Additionally, academic procrastination was often associated with parents employing harsh discipline and strict supervision as well as low levels of emotional support and verbal communication (Zakeri et al., 2013). Conversely, positive parenting styles, such as parents’ moderate concern and understanding for their children emotionally and establishing a democratic family environment, are helpful for individuals to form reasonable time management disposition, and may reduce the degree of academic procrastination of individuals to a certain extent, thus reducing procrastination behavior (Zhiguo et al., 2018). These results emphasize that students’ academic procrastination is closely related to the parenting styles they have experienced. Recently, research has begun to explore the mechanisms underlying these relationships. For example, a recent study found that positive parenting styles (such as compassionate and supportive parenting) not only directly affect the academic performance of college students but also indirectly reduce their procrastination behavior by improving their self-esteem (Batool, 2020). However, such research efforts are still scarce. Little is known about other underlying mechanisms. Especially in China culture, the relationship between academic procrastination and parenting style has not been fully clarified.

H1: We hypothesize that parenting style will directly predict the academic procrastination of nursing students.

### 1.2. Potential mediation of causal attribution

The concept of “attribution” originated from Haider, aiming at understanding the cause of events or explaining the causal relationship of other people’s behaviors (Weiner, 1995). After drawing lessons from Rotter’s theory of location of control, Weiner’s attribution theory proposed four possible behavioral attributions namely: ability attribution, effort attribution, luck attribution and context attribution (Weiner, 1994). Furthermore, Weiner emphasizes the three potential dimensions of locus of causation, stability, and controllability that can be used to categorize any causal attribution (Lee and Hall, 2020). For example, Weiner viewed ability to be internal (locus), stable, and uncontrollable; effort to be internal (locus), unstable, and controllable; luck to be external (locus), unstable, and uncontrollable; and context to be external (locus), unstable, and controllable (Weiner, 2010). Among these three dimensions, the locus of causality has the greatest impact on the student’s academic achievement, with internal locus control related to successful academic achievement, while external locus control is more correlated to failed academic achievement (Lebedina-Manzoni, 2004; Badri Gargari et al., 2011). Individuals’ affective reactions to the causal attribution of academic success or failure can affect their expectations for the future, and thus affect their subsequent behavior (e.g., academic commitment) (Kong et al., 2016), so nursing students’ causal attribution is the key to understanding their academic procrastination. It has been reported that internal attribution was negatively related to academic procrastination, while external attribution was positively related to academic procrastination (Rakes et al., 2013). For example, students with internal attribution style complete learning tasks earlier and have better academic performance (Carden et al., 2004; Houston, 2016). Conversely, students with procrastination have more obvious external attribution style than normal students (Janssen and Carton, 1999). This may be related to the fact that students with internal attribution style believe that academic success comes from their own working ability and effort, and thus they may have better learning input than their peers with external attribution style.

On the other hand, parenting style is an important influencing factor of children’s causal attribution. For example, the study (Georgiou et al., 2016) found that students who often experience rough parental discipline or have a history of foster care had high scores on external attribution, and had problems such as being too sensitive or even aggressive in sexual behavior. However, students who often received parental care and understanding scored higher on internal attribution (Wischerth et al., 2016), which is more conducive to their psychology and health (Li et al., 2022). Besides, parents of children with higher internal attribution were more willing to promote their children’s independence (Carton and Nowicki, 1994). Furthermore, a recent study (Li et al., 2022) found that good parent-child communication not only helps to bring encouragement, confidence and warmth to teenagers, but also helps them to form a positive attribution model to some extent, such as attributing their achievements to their internal factors-hard work. Conversely, overprotective, controlled and rejected parenting styles were significantly associated with the high external attribution (Cohen et al., 2008; Spokas and Heimberg, 2009).

H2: Therefore, this study hypothesizes that causal attribution can be used as a mediating variable between parenting style and academic procrastination of nursing students.

### 1.3. Potential mediation of self-efficacy

Self-efficacy is defined as a belief in a person’s ability to learn or perform behavior at a specific level. Bandura believes that self-efficacy can affect behavior through cognition, motivation, emotion and selectivity (Bandura, 1993). Specifically, high self-efficacy is conducive to promoting individuals’ positive expectations for behavior results. In addition, it can inhibit procrastination by reducing the negative experience of individuals in the process of action. Likewise, self-efficacy also affects the level and persistence of personal efforts to tasks (Bandura, 1977, 1993). People with high self-efficacy are good at actively adapting or changing the environment, striving to overcome difficulties and persisting in tasks for a long time. Therefore, the tendency of academic procrastination is related to the perception of self-efficacy. Previous studies have found that self-efficacy can negatively predict academic procrastination (Klassen et al., 2008; Bakar and Khan, 2016). Furthermore, the results of meta-analysis show that self-efficacy was an important and stable predictor of academic procrastination.

According to social cognitive theory, the formation of self-efficacy is influenced by the expectation, guidance and social support of important people in life (Bandura, 1986), and positive parenting style is helpful to promote the development of individual self-efficacy. Compared with adolescents from autocratic or indulgent parenting styles, children from authoritative parenting styles have higher self-efficacy beliefs (Turner et al., 2009; Tam et al., 2012). Previous studies have found that negative parenting styles (such as autocracy, connivance or non-participation) are not conducive to the formation of children’s self-guidance or self-regulation ability, but these abilities are the basis for the formation of children’s strong self-efficacy and academic success (Diaz, 2005). On the contrary, teenagers who think their parents are warm, democratic and supportive tend to develop positive attitudes and beliefs, so they perform better academically (Hayek et al., 2022). In addition, Masud et al. (2016) found that self-efficacy mediated the relationship between authoritative parenting style and academic achievement.

H3: Therefore, this study hypothesizes that self-efficacy may be the mediating variable between parenting style and academic procrastination.

### 1.4. Relationship between causal attribution and self-efficacy

Individual self-efficacy is also closely related to causal attribution. In attribution theory, the Weiner (1992) emphasizes that the attribution of behavioral success or failure affects its subsequent actions or expectations (such as self-efficacy). Self-efficacy enhanced if success was attributed to internal factors (i.e., effort or ability), but not if success was attributed to external factors (i.e., luck or opportunity) (Bandura, 1993). Bandura emphasized that the evaluation of past success or failure experience will affect self-efficacy, and then affect future performance, which depends on whether the evaluation of past success or failure experience is attributed to internal factors or external factors (Salanova et al., 2012). Previous studies have found that when positive attribution feedback is given, children’s attribution to their own efforts and abilities will increase correspondingly, and their self-efficacy will enlarge correspondingly, while attributing failure to lack of ability will lead to a decline in self-efficacy. Therefore, an individual’s perceived self-efficacy is influenced by his/her evaluation of the past (causal attribution) (Schunk, 1982). Further research shows that causal attribution can also influence motivation and performance through the mediating role of self-efficacy (Cheng and Chiou, 2010).

H4: Therefore, this study hypothesizes that parenting patterns affect academic procrastination through the chain intermediary of causal attribution and self-efficacy.

**FIGURE 1:**
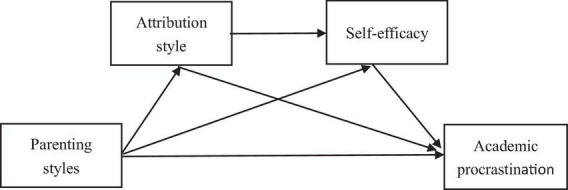
Hypothetical model.

Based on the above findings, we believe that causal attribution style and self-efficacy play a chain intermediary effect between parenting style and academic procrastination. Therefore, we put forward the hypothetical model of this study (Figure 1), so as to explore the influence of parenting style on academic procrastination, and investigate the role of causal attribution style and self-efficacy.

## 2. Materials and methods

### 2.1. Design and participants

A cross-sectional survey was conducted from March to May 2022. The sample of this study comes from nursing undergraduates aged 18–23 years in two four-year undergraduate colleges in Henan Province, China. Participants met the following inclusion criteria: (1) full-time undergraduate nursing students; (2) understand the purpose of the research and volunteer to participate in this study. Exclusion criteria: students who have not completed the questionnaire for various reasons. The final analytic sample included 719 participants, including 152 males (22.3%) and 531 females (77.7%), with an average age of (19.85 ± 1.15) years. Among all participants, more than half were from village and the only-child families accounted for 14.1%. In addition, nearly 90% of nursing students reported that their parents had a harmonious marital relationship in their families.

### 2.2. Procedure

This study was conducted in two undergraduate colleges from March to May 2022. The whole investigation process is organized orderly: first, communicate well with the school in advance; then, after thoroughly explaining the goal and significance of this study to nursing students, questionnaires were delivered to students who volunteered to participate in this study; Third, the questionnaire is short and easy to understand, and students are required to complete it within 15 min. Finally, after eliminating unqualified questionnaires, 683 valid questionnaires were obtained.

### 2.3. Measurements

#### 2.3.1. General demographic data questionnaire

The general demographic information questionnaire assessed the characteristics of the participants, such as age, gender (male, female), residence (town, village), are you an only child? (Yes, no), parent’s marital relations (harmonious, non-harmonious).

#### 2.3.2. Parental bonding instrument (PBI)

We used PBI developed by Parker et al. (2011) to measure the parenting style of parents. PBI is a self-rating scale to evaluate the parents’ attitudes and behaviors of participants in childhood. This study used the Chinese version of the PBI scale revised by Hong-jun et al. (2009), with a total of 46 items and 3 factors: care, encourage independence and control. A 4-point Likert scale was used to score each item. Furthermore, in this study, the dimensions of care and encouraging independence were classified as positive parenting style, while the dimension of control was classified as negative parenting style. In previous studies, the Cronbach’s α of the three dimensions of the Chinese version of PBI scale was 0.736–0.848 (Gao and Zhou, 2011), and it also had good validity (Jiang et al., 2009). In the present study, the Cronbach’s α for the positive and negative parenting dimensions were 0.904 and 0.748, respectively.

#### 2.3.3. The multidimensional-multiattributional causality scale (MMCS)

The MMCS, compiled by Lefcourt et al. (1979) and widely utilized in the research of motivation cognition theory, was used to assess the attribution style of nursing undergraduates. Studies have shown that MMCS scale is suitable for college students (Li et al., 2015; Kong et al., 2016), and it evaluates students’ attribution style from two aspects: academic achievement (24 items) and interpersonal relationship (24 items). In addition, the scale proposed four possible attributions: ability, effort, context, and luck attribution, in which ability and effort belong to internal-controlled attribution, and luck and context belong to external-controlled attribution. Since academic procrastination is mainly related to academic achievement, this study only focuses on the academic achievement part (24 items). Each item was scored with Likert scale of 5 (0 = disagree, 4 = agree). Cronbach’s α of this scale is 0.815. Additionally, it also has good structural validity (Powers and Rossman, 1983) and convergent validity (Powers et al., 1985). In this study, the Cronbach’s α of the scale is 0.750.

#### 2.3.4. General self-efficacy scale (GSEC)

GSEC is used to measure a person’s sense of efficacy in coping with everyday situations and adapting to stressful life events (Schwarzer and Jerusalem, 1995). A total of 10 entries were each scored using 4-point Likert, and higher scores indicating higher self-efficacy. The Cronbach’s α was 0.871 in this study.

#### 2.3.5. Aitken procrastination inventory (API)

The API is a self-rating scale designed by Aitken (1982) in 1982, which is used to evaluate long-term persistent procrastination among college students. It consists of 19 items, of which 9 topics are scored backwards. The scale adopts five-point scoring method, and the higher the score, the more serious the students’ procrastination behavior. The reliability and validity of the Chinese version of the scale in Chinese college students are in line with psychometric criteria (Xiaoli et al., 2008). In this sample, the Cronbach’s α of the scale is 0.852.

### 2.4. Statistical analysis

Statistical analysis was performed using IBM SPSS statistics 25.0 and Amos26.0. First, the mean and standard deviation were used to describe participants’ scores of Parenting styles, causal attribution, self-efficacy and academic procrastination. Second, we used Harman’s single factor test to test the common method bias from self-reported data. Thirdly, all continuous variables are tested for normality. If the data was normally distributed, we used Pearson correlation analysis to test the correlation between variables. Otherwise, Spearman correlation analysis was used. Finally, AMOS26.0 was used to construct structural equation model for mediating effect test. The threshold for all variables’ significance was set at α = 0.05. In accordance with the mediation effect test procedure (Wen and Ye, 2014), we estimated structural model with maximum likelihood method and used χ^2^/df comparative fitting index (CFI), goodness of fit index (GFI), Tucker-Lewis index (TLI), increasing fitting index (IFI), approximated root mean square error (RMSEA) to estimate the fitting degree of the model. For large sample sizes, the threshold value of χ^2^/df is between 3 and 5 were acceptable (Lefcheck, 2016). Furthermore, we used CFI > 0.90, GFI > 0.9, TLI > 0.9, IFI > 0.9, and RMSEA < 0.08 as an indicator for acceptable fit between models (Hu and Bentler, 1999; Byrne, 2016). Then, we used the bootstrapping method to test for indirect effects. This method has the advantage that it can still be used when the data does not obey the normal distribution (Hayes and Preacher, 2010). In bootstrapping, if the 95% confidence interval of the standardized path coefficient does not contain 0, the mediation effect is significant.

### 2.5. Ethics statement

This research has been approved by XXX (ID number: 20220107001). Before starting this investigation, the participants signed the informed consent form, and were told that they could choose not to participate. In order to ensure anonymity, we did not collect student names or other identifiers.

## 3. Results

### 3.1. Common method bias test

With data collected through self-report, common methodological bias problems may arise in this study (Podsakoff et al., 2003). Even if necessary control is carried out in the measurement process, for example, participants fill in the report anonymously and some questions are expressed in reverse (Hao, 2004). To ensure the rigor of this study, Harman’s single factor test was used to test for common method bias. Results showed a total of 25 factors with eigenvalues greater than 1, explaining 60.7% of the variance, with the first factor explaining a variance of 15.3%, much less than the threshold value of 40% (Podsakoff et al., 2003). Thus, there is no serious common method bias in this study.

### 3.2. Correlation analysis of parenting style, causal attribution, self-efficacy, and academic procrastination

Table 1 shows the average and standard deviation of parenting style, causal attribution, self-efficacy and academic procrastination scores, and correlation coefficient between among the variables. Pearson correlation analysis showed that SE was positively associated with IA (*r* = 0.381, *p* < 0.01) and negatively associated with EA (*r* = –0.377, *p* < 0.01). Furthermore, we also found a significant negative relationship between AP and PPS, IA, and SE (*r* = –0.350, *p* < 0.01; *r* = –0.349, *p* < 0.01; *r* = –0.454, *p* < 0.01), and a significant positive relationship with NPS and EA (*r* = 0.402, *p* < 0.01; *r* = 0.447, *p* < 0.01).

**TABLE 1:** Descriptive statistics and correlations between study variables (*N* = 683).

Variable	Mean ± SD	1	2	3	4	5	6
PPS	1.97 ± 0.42	1					
NPS	1.42 ± 0.46	−0.550**	1				
IA	3.36 ± 0.49	0.383**	−0.319**	1			
EA	2.94 ± 0.58	−0.300**	0.319**	0.040	1		
SE	2.72 ± 0.61	0.342**	−0.323**	0.381**	−0.377**	1	
AP	2.81 ± 0.60	−0.350**	0.402**	−0.349**	0.447**	−0.454	1

M, mean; SD, standard deviation; PPS, positive parenting style; NPS, negative parenting style; IA, internal attribution; EA, external attribution; SE, self-efficacy; AP, academic procrastination.

**P < 0.01.

### 3.3. Measuring model

Validating factor analyses were needed to test the measurement model prior to testing for mediating effects. We developed a measurement model with 4 latent variables (parenting style, attributional style, self-efficacy, and academic procrastination) and 24 observed variables. We determined that the measurement model developed was a good fit [χ^2^ (161) = 577.931; GFI = 0.925, CFI = 0.915; NFI = 0.886; SRMR = 0.044; RMSEA = 0.062]. In addition, we found that all observed standardized loadings of each indicator on the corresponding factors were significant (*p* < 0.05 between 0.310 and 0.618).

According to the previous theoretical basis and the results of the correlation matrix, the parenting style is used as the independent variable of this study, the academic procrastination style is used as the dependent variable, and the causal attribution and self-efficacy are used as the mediating variables to construct the model. According to two paths: positive parenting style → internal attribution → self-efficacy → academic procrastination and negative parenting style → external attribution → self-efficacy → academic procrastination, we constructed 2 latent variable mediation models.

### 3.4. The mediating effect of causal attribution and self-efficacy

#### 3.4.1. Mediating analysis of internal attribution and self-efficacy between positive parenting styles and academic procrastination

**FIGURE 2:**
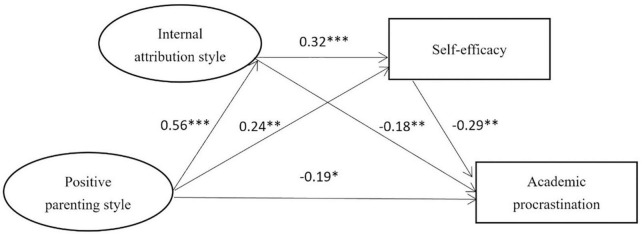
The model of internal attribution style and self-efficacy as mediators between positive parenting style and academic procrastination. *, **, *** denotes *p* < 0.05, *p* < 0.01, *p* < 0.001, respectively.

The model (standardized path coefficient) mediated by internal attribution and self-efficacy is shown in Figure 2. The model 1 fits well: χ^2^/df = 4.910 < 5, CFI = 0.980, GFI = 0.988, AGFI = 0.950, TLI = 0.939, IFI = 0.980, and RMSEA = 0.08. Positive parenting styles positively predicted internal attribution and self-efficacy, and negatively predicted academic procrastination (β = 0.56, *P* < 0.001; β = 0.24, *P* < 0.01; β = –0.19, *P* < 0.05); Internal attribution positively predicted self-efficacy (β = 0.32, *P* < 0.001); Internal attribution and self-efficacy both negatively predicted academic procrastination (β = -0.18, *P* < 0.01; β = -0.23, *P* < 0.01). Using ML method to test the mediating effect, the 95% CI of each mediating path does not contain 0, and the mediating effect is significant (Table 2). Internal attribution and self-efficacy accounted for 45.34% of the total effect, and played a partial mediating role.

**TABLE 2:** Bootstrap analysis of the mediating model.

	Effect	Path	Effect	Bootstrap SE	95% CI	Relative mediating effect (%)
Model 1	Total	PPS → AP	−0.41			
	Direct	PPS → AP	−0.19	0.06	−0.30, −0.08	46.34
	Indirect	PPS → IA → AP	−0.10	0.03	−0.18, −0.04	24.39
		PPS → SE → AP	−0.07	0.02	−0.11, −0.03	17.07
		PPS → IA → SE → AP	−0.05	0.01	−0.09, −0.03	12.20
Model 2	Total	NPS → AP	0.40			
	Direct	NPS → AP	0.20	0.04	0.12, 0.28	50.00
	Indirect	NPS → EA → AP	0.12	0.02	0.07, 0.17	30.00
		NPS → SE → AP	0.05	0.01	0.03, 0.08	12.50
		NPS → EA → SE → AP	0.03	0.01	0.02, 0.05	7.50

PPS, positive parenting style; IA, internal attribution; SE, self-efficacy; AP, academic procrastination; PPS, positive parenting style; EA, external attribution.

#### 3.4.2. Mediating analysis of external attribution and self-efficacy between negative parenting styles and academic procrastination

**FIGURE 3:**
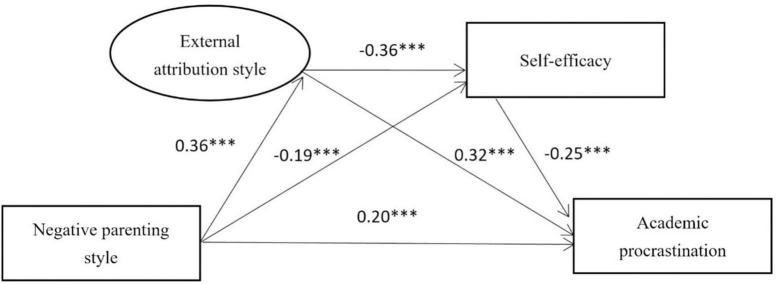
The model of external attribution style and self-efficacy as mediators between negative parenting style and academic procrastination. *** indicates *p* < 0.001.

The model (standardized path coefficient) mediated by external attribution and self-efficacy is shown in Figure 3. Model 2 fits well: χ^2^/df = 4.261 < 5, CFI = 0.992, GFI = 0.995, AGFI = 0.963, TLI = 0.962, IFI = 0.992, and RMSEA = 0.069. Negative parenting styles positively predicted external attribution and academic procrastination; and negatively predicted self-efficacy (β = 0.36, *P* < 0.001; β = 0.20, *P* < 0.001; β = –0.19, *P* < 0.001); External attribution negatively predicted self-efficacy (β = -0.36, *P* < 0.001); External attribution positively predicted academic procrastination (β = 0.32, *P* < 0.001) and self-efficacy negatively predicted academic procrastination (β = -0.25, *P* < 0.001). Using ML method to test the mediating effect, the 95% CI of each mediating path does not contain 0, and the mediating effect is significant (Table 2). External attribution and self-efficacy accounted for 50.00% of the total effect, and played a partial mediating role.

## 4. Discussion

### 4.1. Influence of parenting styles on academic procrastination

This study showed that positive and negative parenting styles can negatively and positively predict academic procrastination of nursing undergraduates respectively **(Hypothesis 1)**. This suggests that the more parents care and encourage nursing students, the more likely they are to actively participate in learning and have less academic procrastination, while nursing students who experience negative parenting style are opposite. This is consistent with existing research (Chen et al., 2015; Won and Yu, 2018). Parenting style, as an integral part of the family micro-system, has a significant impact on teens’ personality, behavior, attitude, and many other aspects (Thimm, 2010; Jones et al., 2012), while academic procrastination, as a behavior attitude, is also affected by the family environment, especially parenting style (Luo Yun and Zhenhong, 2016). Authoritarian parenting style, such as strict control and supervision, neglecting children’s attitudes, is not conducive to the formation of individual healthy psychology, hinders individual personality development, and easily lead to academic procrastination (Zakeri et al., 2013). Especially in East Asia, parents are strict with their children’s academic performance and control their children seriously (Pomerantz and Wang, 2009). Students who are excessively interfered and controlled by their parents have low adaptability and are more likely to feel frustrated and helpless (Won and Yu, 2018). In addition, it is easy to have a negative attitude toward study, and even a rebellious attitude, which is manifested as academic procrastination. In contrast, students living in a warm and constructive family atmosphere tend to gain understanding and support and face difficulties and setbacks in learning with a positive attitude, resulting in less academic procrastination (Vahedi et al., 2009).

### 4.2. Mediating effect of causal attribution

This study found that positive parenting style not only negatively affects learning procrastination directly, but also indirectly negatively affects academic procrastination through the mediating effect of internal attribution. To the contrary, negative parenting style not only directly and positively affects academic procrastination, but also indirectly positively affects academic procrastination through the mediating effect of external attribution (**Hypothesis 2**). This indicates that positive parenting style is beneficial to develop students’ internal attribution style, making them believe that personal efforts and abilities are related to learning success or failure, so as to actively engage in learning and reduce academic procrastination. However, negative parenting style easily led to the formation of students’ external attribution, which makes them think that academic success or failure depends on external factors (e.g., luck, opportunity), and lacks learning motivation, resulting in academic procrastination. Causal attribution is mainly restricted by external environmental factors, of which the family environment is especially important for individual cognitive development (Li and Qian, 2002). For example, Chinese adolescents with less parental attention and more rejection and punishment by their parents reported more negative attribution styles (Xu, 2000). Besides, encouraging independent parenting will promote children’s independent development and form a high internal attribution. However, negative parenting styles, such as doting and controlling, will keep children in a restricted state for a long time, like to be controlled by others and easily form external attribution style (Keshavarz et al., 2014). Additionally, the attribution style reflects the students’ self-perception and can influence their expectations and beliefs about their abilities, and subsequently manipulate their behavioral motivation (Badri Gargari et al., 2011). For instance, Brownlow et al. (Brownlow and Reasinger, 2012) found that highly procrastinating undergraduates attribute test-taking success to external, erratic factors and see themselves as contributing little to academic achievement. However, low-procrastinators are prone to attribute their academic success to effort. In this study, attribution style, as a cognitive style, mediated the relationship between parenting style and nursing undergraduates’ academic procrastination: it was not only affected by parenting style, but also significantly predicted students’ academic procrastination level.

### 4.3. Mediating effect of self-efficacy

Further research in this study found that, besides causal attribution, self-efficacy also partially mediated the impact of parenting style on academic procrastination **(Hypothesis 3)**. Specifically, higher self-efficacy was related to lower academic procrastination, which was consistent with earlier findings (Klassen et al., 2008). Self-efficacy plays a significant role in individual psychology, which will have a huge impact on individual behavior, beliefs, and achievements, and can promote individual mental health (Abdel-Khalek and Lester, 2017). It is difficult to avoid setbacks and pressures in the process of completing their studies. High self-efficacy students have stronger anti-interference ability and are less negatively affected when faced with high pressure and anxiety. Therefore, students with high self-efficacy have good faith in completing academic tasks (Chow, 2011), thus reducing the tendency to procrastinate. However, students with low self-efficacy will fall into a vicious cycle of procrastination in their study (Waschle et al., 2014). Therefore, cultivating students’ self-efficacy is an important way to improve their learning input and reduce academic procrastination.

### 4.4. Chain mediation effect between causal attribution and self-efficacy

Some researchers have suggested that causal attribution can indirectly influence learning attitudes and behaviors by affecting self-efficacy. Feedback that attributes success or failure to internal factors (e.g., effort) can increase students’ self-efficacy, while conversely attributing failure to external factors (e.g., task difficulty) results in lower expectations for the future (Schunk, 1982; Su et al., 2021). This statement is supported by this study. This study found that causal attribution was strongly correlated with self-efficacy, which constitute the intermediate link of positive(negative) parenting style → internal attribution (external attribution) → self-efficacy → academic procrastination **(Hypothesis 4)**. Self-efficacy and attribution theorists believe that learners’ self-efficacy level and their attribution style of success and failure will affect their efforts and persistence, and ultimately motivation and achievement (Bandura, 1986; Weiner, 2000), so some types of attribution may be better. A previous study found that nursing students who are accustomed to more ability attribution and effort attribution have a positive attitude toward their careers, and think they have the ability to change the unfavorable career environment (Kong et al., 2016). Thus, the best attribution for causing adaptive behavior is to attribute academic procrastination to self-internal factors. In this way, nursing students usually think that they should be held accountable for event outcomes, thus actively increasing their investment in learning and helping to improve their professional confidence. Therefore, it is necessary for nursing educators to conduct attribution training (Hall et al., 2007) for nursing students with academic procrastination, and should focus on cultivating their ability attribution and effort attribution.

### 4.5. Limitations and future directions

This study has some limitations. First, this study was only conducted among undergraduate nursing students in 2 universities in Henan Province, China, which may limit the generalizability of the results. Secondly, this study is a cross-sectional study, which only examines the relationship between variables at a certain time point, and cannot infer a clear causal relationship. Third, the data collection adopts the way of self-reporting, and the results may have subjective deviation. It is suggested that a multi-center longitudinal study can be carried out in future research, and a variety of evaluation methods can be combined, such as combining self-evaluation with peer evaluation, teacher or parent evaluation, to further explore the mechanism of action among variables, so as to clarify the reasons for academic procrastination of nursing undergraduates.

## 5. Conclusion

This study investigated the parenting styles, attribution style, self-efficacy, and academic procrastination of undergraduate nursing students. In this study, a chain mediation model was constructed from the perspective of individual psychological quality to explore the process and mechanism of parenting style affecting the academic procrastination of nursing undergraduates. This study found that parenting styles can directly predict the academic procrastination of undergraduate nursing students, with a significant negative relationship between positive parenting style and academic procrastination, and that between negative parenting style and academic procrastination. Furthermore, parenting styles can also indirectly predict undergraduate nursing students’ academic procrastination through mediation of attribution style and self-efficacy. Attribution style and self-efficacy can influence the relationship separately, and they can also act as chain intermediaries. This result not only enriches the theoretical explanation and empirical evidence of the procrastination mechanism, but also provides more options for substantive intervention in undergraduate nursing students’ academic procrastination.

## 6. Implications

The results of this study have some implications for nursing student education. First of all, the effect of positive parenting on academic procrastination of nursing undergraduates suggests that parents should pay attention to parenting methods, avoid using negative methods (such as control, rejection and negation), and adopt more positive parenting methods (such as caring, understanding and encouragement) to interact with students, so as to promote their learning input and reduce academic procrastination. Secondly, attribution style and self-efficacy of nursing students play a chain intermediary role between parenting styles and academic procrastination. It is suggested that educators should change students’ attribution style in the teaching process, cultivate students’ belief in self-control of academic procrastination, and enhance students’ self-confidence in solving learning difficulties, thus effectively reducing academic procrastination. Although attribution styles are relatively stable, it still has a certain plasticity. Nursing teachers can guide students to form adaptive attribution styles through attribution training, deepen their awareness and beliefs about academic failure and success, and help to reduce students’ stigma and negative emotions about academic procrastination to improve their motivation for future achievement.

## Data availability statement

The original contributions presented in this study are included in the article/supplementary material, further inquiries can be directed to the corresponding author.

## Ethics statement

The studies involving human participants were reviewed and approved by the Review Committee of Psychological Research Ethics Review Organization of Henan Key Laboratory of Psychology and Behavior (ID number: 20220107001). The patients/participants provided their written informed consent to participate in this study. Written informed consent was obtained from the individual(s) for the publication of any potentially identifiable images or data included in this article.

## Author contributions

YL and WD were responsible for the study design and critical revision of the manuscript. YL wrote the first draft of the manuscript. WD, HT, XG, SW, and CC were responsible for analysis and interpretation of data. GL provided the statistical expertise. CC directed all the work. All authors have read and agreed to the published version of the manuscript.
